# Microbiological quality of air in free-range and box-stall stable horse keeping systems

**DOI:** 10.1007/s10661-018-6644-0

**Published:** 2018-04-07

**Authors:** Katarzyna Wolny-Koładka

**Affiliations:** Department of Microbiology, University of Agriculture in Cracow, Mickiewicza Ave 24/28, 30-059 Cracow, Poland

**Keywords:** Horses, Air sampling, Bacteria, Fungi, Box-stall stable, Free-range

## Abstract

The aim of this study was to assess the microbiological quality of air in three horse riding centers differing in the horse keeping systems. The air samples were collected in one facility with free-range horse keeping system and two with box stalls of different sizes. The samples were collected over a period of 3 years (2015–2017), four times per year (spring, summer, autumn, winter) to assess the effect of seasonal changes. The prevalence of aerobic mesophilic bacteria, mold fungi, actinomycetes, *Staphylococcus* spp., and *Escherichia coli* was determined by the air collision method on Petri dishes with appropriate microbiological media. At the same time, air temperature, relative humidity, and particulate matter concentration (PM_10_, PM_2.5_) were measured. It was found that the horse keeping system affects the occurrence of the examined airborne microorganisms. Over the 3-year period of study, higher temperature and humidity, as well as particulate matter concentration—which notoriously exceeded limit values—were observed in the facilities with the box-stall system. The air sampled from the largest horse riding center, with the largest number of horses and the box-stall system of horse keeping, was also characterized by the heaviest microbiological contamination. Among others, bacteria from the following genera: *Staphylococcus* spp., *Streptococcus* spp., *Bacillus* spp., and *E. coli* and fungi from the genera *Aspergillus*, *Fusarium*, *Mucor*, *Rhizopus*, *Penicillium*, *Trichothecium*, *Cladosporium*, and *Alternaria* were identified in the analyzed samples.

## Introduction

Stables and other livestock buildings are not only a place for breeding and keeping animals, but also a work environment for people. Sanitary condition of air in stables has a direct effect on the health and well-being of people working with horses and the animals themselves. The causes of pollutant accumulation include frequent in such places high organic dust concentration, as well as small airflow inside rooms where animals are kept. In livestock buildings, air pollution originates mainly from animals, their feces, feed, and litter (Lloyd et al. 2003). Bioaerosol-forming microorganisms of livestock rooms are characterized by opportunistic virulence, but in cases of reduced immunity, periodically occurring in humans or animals, the air may be the source of pathogenic strains that cause zoonoses and epizootic diseases. Airborne microorganisms that may pose an epidemiological threat and can be found in livestock premises include enteropathogenic strains of *Escherichia coli* and other bacteria of fecal origin as well as *Staphylococcus* spp., *Pseudomonas* spp., *Acinetobacter* spp., and *Erwinia* spp., that can dwell in the litter or settle on the dust particles and together pose a health threat in the form of bioaerosol (Zucker et al. 2000; Elfman et al. 2009; Witkowska et al. 2012; Korzekwa et al. 2015). Mold fungi, including *Cladosporium* spp., *Alternaria* spp., *Aspergillus* spp., *Penicillium* spp., *Fusarium* spp.; their spores and metabolites; mycotoxins, and allergenic actinomycetes also pose a major threat; their presence in the air of stables negatively affects the health of humans and animals (Korzekwa et al. 2015). Toxicological hazards occur especially in those livestock rooms where negligence leads to the accumulation of feces, contaminated feed, dirt, and excessive humidity. Often, causes of poor sanitary condition include technological defects (e.g., inadequate material and construction solutions, lack of ventilation), improper operation, or negligence of repairs of facilities for animals (Duchaine et al. 2000; Chang et al. 2001). It should be emphasized that horses, their lovers, and caregivers spend a lot of time in stables and may be exposed to the harmful bioaerosol, which in consequence can result in lung diseases (Elfman et al. 2009). Particulate matter (PM) affects the abundance of bioaerosol-forming microorganisms as it is the main transport agent for biological particles (Frąk et al. 2014). Standards containing limit concentrations of PM_10_ and PM_2.5_ in Poland are included in the Regulation of the Minister of Environment of 24 August 2012 on the levels of certain substances in the air (Regulation 2012). The surface of particulate matter fragments contains microorganisms, which altogether penetrate into the respiratory system. Biological particles of diameters smaller than 7 μm (respirable fraction) are a significant problem; those with a diameter of 4.7–7 μm deposit mainly in the nasopharynx; particles of a diameter 3.3–4.7 μm reach the trachea and primary bronchi; 1.1–3.3 μm particles reach the secondary and terminal bronchi, while those smaller than 1.1 μm may pass into alveoli (Górny and Dutkiewicz 2002). Bioaerosol particles smaller than 5 μm may stay suspended in the air, while the larger ones undergo sedimentation (Chmiel et al. 2015).

In horses, as in humans, respiratory diseases are a huge problem. After orthopedic disorders, they are the second most important cause of exercise dysfunction in these animals. In young individuals, the most common are lung and upper respiratory tract diseases caused by infectious agents such as bacteria, fungi, actinomycetes, viruses, and toxins of microbial origin. In horses older than 7 years of age, the most common causes of this type of diseases are allergic conditions. RAO (recurrent airways obstruction) previously called COPD (chronic obstructive pulmonary disease), which is a disorder similar to human asthma, is the lung disease most commonly encountered in both recreational and sports horses. In the course of this disease, the horse wears quickly, coughs, and has a white discharge from nostrils (Witkowska et al. 2012).

At present, we have four main horse keeping systems: box stalls, tethering, run pen, and free-range (Waran 2002). The horse keeping system depends on the race, age, sex, and destination of the horses as well as the possibilities of the facility. In this study, concentration of bioaerosol was determined in the horse riding centers that differ in the horse keeping system, i.e., box-stall and free-range types; hence, these two types are described below. The most popular type of horse keeping is the box-stall system that restricts both movement and contact with other animals. It is often used, particularly in the case of noble breeds, or especially valuable individuals such as foals, stallions, sports horses, and aggressive or sick animals. It is also a very convenient and cost-effective way of keeping horses that do not require frequent supervision, while at the same time provides easy access to the animals. However, it is extremely important that the box stalls meet certain parameters with respect to microclimate, construction technology, and parameters affecting animal welfare. It should be remembered that this is not the optimal system for horses, as those animals have a strong herd instinct. On the other hand, the free-range system which provides horses with the possibility of unrestricted movement and contact with other animals is the most suited to the nature of horses. Mutual contacts within the flock enable to establish the hierarchy and to keep animals in peace. It is important to select the appropriate individuals, eliminating aggressive and malicious ones. Although access to horses on the pasture may be difficult and at the same time their observation is necessary, economically, it is an extremely cost-effective type of horse keeping. The free-range system is ideal for Hutsul horses that are resistant to adverse climatic condition and have a strong herd instinct (Waran 2002). However, regardless of the horse keeping system, the conditions in which they stay must be optimal enough and adapted to the needs of animals in order to maintain them in good health and condition.

Animal welfare and hygiene of employees of the horse riding centers, as well as adequate cleanliness at the stables, are a way to prevent the spread of pathogenic microorganisms. Therefore, finding increased concentrations of particulate matter and the number of selected microbial groups in the tested air should encourage taking measures in order to eradicate harmful microorganisms. Currently, there are no uniform regulations determining acceptable concentrations of microorganisms in the air of livestock premises (Gołofit-Szymczak and Górny 2010). In addition, research on this subject usually concerns pig, cattle, or chicken farms where animals are kept in high density (Witkowska et al. 2012). In stables, microbiological contamination may reach different levels depending on many factors such as construction systems, type of ventilation, size of premises, density of stocking, microclimatic conditions, and type of litter. For that reason, studies on the concentration of airborne microorganisms in stables should be conducted in order to determine the potential health risks for both humans and horses, often extremely valuable for their caregivers (Witkowska et al. 2012).

The aim of the conducted study was to assess the microbiological quality of air in stables with different sizes of box stalls and in the stable with the free-range horse keeping system. On this basis, it was expected to determine how the horse maintenance system affects the concentration and composition of bioaerosol.

## Material and methods

### Sampling points

The microbiological quality of air was investigated in three, differing in the type of horse keeping systems, horse riding centers located in the Lesser Poland voivodeship (Poland). Horse Riding Center Pegaz in Kraków (OJK Pegaz) is an example of a small stable, with seven closed and eight boxes opening to the outside. There are 13 horses in the facility, including Shetland pony, half-blood noble horse, and Wielkopolski horse, and the remaining two are horses owned by private people. The horses kept in the center are recreational horses, and apart from private horses, there is no rotation. The sampling sites were located in five points. Points no. 1 and 2 were located inside the closed stable, points no. 3 and 4—in the open box stalls—and the point no. 5 in front of the stable, outdoors (control point). The Horse Riding Club Szary in Michałowice (KJK Szary) is one of the largest and most modern stables in Poland; it has a total of c.a. 100 closed boxes. KJK Szary keeps recreational and sports horses of many breeds, including Shetland pony, half-blood noble horse, Wielkopolski horse, and Belgian hot-blooded horse, and the facility also runs a guesthouse for horses, hence the large rotation of animals. Sampling sites were located in 10 points. Points no. 1 to 9 were located inside the stable, whereas point no. 10 (control) was situated outdoors, in front of the stable. The sampling sites in OJK Pegaz and KJK Szary were evenly distributed so that the air in the stable could be analyzed in a representative way. In both stables (OJK Pegaz and KJK Szary), a gravity ventilation system was used. The Hutsul Pony Stud Farm in Nielepice (SKH Nielepice) is the only one that runs the non-stable animal husbandry. The horses stay in the open air all year round. The Hutsul horses are kept on pastures in two separate herds (10 mares, 18 rolls, and 2 stallions) and only use shelters in wooden sheds without doors. Sampling sites were located in four points relevant for the operation of the stud (1—roof for riders; 2—saddle room; 3—roof for horses; 4—paddock). The number of horses in all three riding centers remained the same during the study period.

### Air sampling, microbiological analysis

Air samples were collected every 4 months (spring—April; summer—July; autumn—October; winter—January), for 3 years, resulting in 12 sampling campaigns. Air for microbiological testing was sampled in triplicates using the MAS-100 (Merck, Switzerland) air sampler. Each time from the height of 1.5 m, 100 l of air was collected over 1 min, in accordance with the requirements specified in the Polish Standard (PN-Z-04008-08 1989). The number of microorganisms was determined by culturing on the following microbiological media: mesophilic bacteria (Trypticasein Soy Lab Agar, BTL, Poland), mold fungi (Malt Extract Agar, BTL, Poland), actinomycetes (Actinomycete Isolation Lab Agar, BIOCORP, Poland), and staphylococci (Chapman agar, BTL, Poland). In addition, the presence of *Escherichia coli* in the air of the tested points was determined using the chromogenic medium TBX (Tryptone Bile X-glucuronide, BTL, Poland). After sampling, the cultures were incubated at the following conditions: mesophilic bacteria and staphylococci 37 °C, 48 h; mold fungi 28 °C, 3–5 days; actinomycetes 28 °C, 5–7 days; and *E. coli* 44 °C, 48 h. At each sampling point, air temperature and relative humidity (Kestrel 4000, Nielsen-Kellerman, USA) as well as the concentration of particulate matter (PM_10_, PM_2.5_, sampling time—1 min; sampling interval—1 min.) (dust meter DustTrak, TSI) were measured. After incubation, the characteristic colonies of tested microorganisms were counted and the results were given as the number of colony forming units per cubic meter of air (CFU·m^−3^), with the consideration of the positive hole correction table as recommended by the air sampler manufacturer (Operator’s Manual MAS-100TM professional Microbial Air Monitoring System for the Microbiological Testing of Air n.d.). Preliminary identification of airborne microorganisms was also performed using diagnostic keys (Domsch et al. 1980; Holt 1994). During the entire study period, it was checked whether the horses participating in the experiment remained in good condition and were not subjected to pharmacological treatment.

### Statistical analysis

The statistical analysis was conducted in Statistica v. 12.5 software (StatSoft, US). A two-way ANOVA test was performed to determine the statistical significance of temporal and spatial variation in the bioaerosol. Pearson’s correlation coefficient *r* was calculated between the abundance of airborne microorganisms and microclimatic parameters (temperature, relative humidity, PM_10_, and PM_2.5_).

## Results and discussion

Considering the fact that microclimatic conditions that prevail in livestock premises favor the presence of many groups of microorganisms, their large number and diversity should not be surprising (Korzekwa i in. 2015). On the other hand, the presence of pathogenic bacteria and fungi and their metabolites can pose a serious threat to the health of people and animals (Wolny-Koładka and Malina 2017). Based on a 3-year study on microbial contamination of air in the non-stable and box-stall system of horse keeping, the presence of bacteria from the following genera was found in the analyzed material: *Staphylococcus*, *Streptococcus*, *Bacillus*, *Micrococcus*, *Diplococcus*, and *Sarcina*. The following genera were identified in the case of fungi: *Aspergillus*, *Fusarium*, *Mucor*, *Rhizopus*, *Penicillium*, *Trichothecium*, *Cladosporium*, and *Alternaria*. As shown by other authors, these are microorganisms typical for the environment of livestock premises (Dutkiewicz et al. 1994; Zucker et al. 2000; Witkowska et al. 2012). On the basis of the environmental interview, it was not found that the horses, included in the experiment, were sick or treated pharmacologically in the study period. Due to the lack of applicable standards in Poland that would determine the limit concentrations of airborne microorganisms in livestock premises, the results obtained in this study were referred to the criteria proposed by the National Institute of Hygiene (PN-89/Z-04111/02 1989; PN-89/Z04111/03 1989) presented in Table 1, and—in the case of *Staphylococcus* spp. and *E. coli*—to the papers by other authors (Dutkiewicz et al. 1994; Zucker et al. 2000; Korzekwa et al. 2015; Budzińska et al. 2016).

**Table 1:** Limit values (CFU·m^−3^) concerning microbial air contamination

Mesophilic bacteria	Mold fungi	Actinomycetes	Level of air contamination
< 1000	3000–5000	< 10	No contamination
1000–3000	5000–10,000	10–100	Moderate contamination*
> 3000	> 10,000	> 100	Heavy contamination**

Own study based on Polish Standard (PN-89/Z-04111/02 1989; PN-89/Z04111/03 1989)

Single (*) and double asterisk (**) interpretation of data is in Tables 2, 3, and 4

The assessment of dust concentration was based on the limits set by the Regulation of the Minister of the Environment of 24 August 2012 on the levels of certain substances in the air, which contain the limit values for the particulate matters PM_10_ and PM_2.5_ (Regulation 2012).

The results of studies concerning mean concentrations of individual groups of bioaerosol-forming microorganisms are presented in Tables 2, 3, and 4.

**Table 2:** Mean number (CFU·m^−3^) of airborne microorganisms—OJK Pegaz

Season	Sampling point					
	1	2	3	4	5	Mean
Mesophilic bacteria						
Spring	2559* c	2412* c	1357* ab	2868* c	354 ab	1910
Summer	3923** c	4367** c	1914* ab	3041** c	535 ab	2756
Autumn	1980* abc	2450* c	847 ab	2167* bc	78 a	1504
Winter	1916* ab	2205* bc	75 a	94 a	28 a	864
Mold fungi						
Spring	1150 ab	1031 ab	1494 ab	460 ab	247 ab	876
Summer	1414 ab	1544 b	827 ab	1528 ab	696 ab	1202
Autumn	1890b ab	2420 b	512 ab	107 ab	70 ab	1000
Winter	586 ab	358 ab	74 ab	62 a	87 ab	233
Actinomycetes						
Spring	552** a	701** a	28* a	57* a	16* a	271
Summer	86* a	35* a	6 a	75* a	4 a	41
Autumn	0 a	46* a	0 a	41* a	0 a	17
Winter	29* a	16* a	15* a	2 a	0 a	13
*Staphylococcus* spp.						
Spring	2152 ab	1163 ab	1534 ab	2479 ab	76 a	1481
Summer	1534 ab	3752 b	1706 ab	1992 ab	202 a	1837
Autumn	2037 ab	2617 ab	225 a	2204 ab	0 a	1417
Winter	820 ab	890 ab	69 a	56 a	16 a	370
*E. coli*						
Spring	25 ab	15 ab	13 ab	31 ab	18 ab	20
Summer	31 ab	47 b	13 ab	8 ab	1 a	20
Autumn	0 a	0 a	0 a	0 a	0 a	0
Winter	0 a	0 a	0 a	0 a	0 a	0

The different letters within a column indicate a significant difference at *p* < 0.05 according to Tukey’s test

*Moderate air contamination described in Table 1

**Heavy air contamination described in Table 1

**Table 3:** Mean number (CFU·m^−3^) of airborne microorganisms—KJK Szary

Season	Sampling point										
	1	2	3	4	5	6	7	8	9	10	Mean
Mesophilic bacteria											
Spring	2056* abc	2200* abc	2040* abc	2625* bc	3300** c	3442** c	2497* bc	1954* abc	3148** bc	278 ab	2354
Summer	3178** c	2821* bc	1951* abc	4113** c	3497** c	4328** c	2999* bc	3521** c	2210* abc	246 ab	2886
Autumn	1790* abc	1983* abc	1283* abc	3357** c	230 ab	2850* bc	3096** bc	3540** c	2061* abc	133 a	2032
Winter	1957* abc	1050* abc	2467* abc	2406* abc	2320* abc	2271* abc	2227* abc	2110* abc	2987* bc	160 ab	1995
Mold fungi											
Spring	2080 abc	1062 abc	1387 abc	1944 abc	1892 abc	3078 c	2954 bc	3144 c	3039 bc	1399 abc	2198
Summer	1666 abc	2082 abc	2364 abc	1487 abc	1142 abc	1957 abc	2002 abc	2475 abc	1584 abc	914 abc	1767
Autumn	3023 bc	3491 c	2310 abc	2220 abc	490 ab	2799 bc	3427 c	3140 c	2351 abc	1177 abc	2443
Winter	556 ab	806 abc	2067 abc	1236 abc	1890 abc	2588 abc	813 abc	477 ab	2028 abc	253 a	1271
Actinomycetes											
Spring	23* a	28* a	10 a	72* a	102** a	102** a	111** a	141** a	169** a	12* a	77
Summer	1803** bc	2361** c	648** ab	1221** abc	699** ab	2468** c	310** a	704** ab	321** a	39* a	1057
Autumn	0 a	0 a	0 a	0 a	0 a	0 a	0 a	20* a	0 a	0 a	2
Winter	67* a	36* a	142** a	426** ab	250** a	266** a	70* a	49* a	34* a	0 a	134
*Staphylococcus* spp.											
Spring	1960 abc	1882 abc	1760 abc	1178 abc	2646 abc	3421 c	2145 abc	3570 c	2556 abc	329 ab	2145
Summer	3134 abc	3246 bc	1787 abc	3671 c	2940 abc	3407 c	3167 bc	3053 abc	4119 c	632 abc	2916
Autumn	1427 abc	2700 abc	1760 abc	943 abc	248 a	2163 abc	2857 abc	3190 bc	977 abc	533 abc	1680
Winter	1157 abc	943 abc	1730 abc	2370 abc	2557 abc	2020 abc	2198 abc	1580 abc	921 abc	378 abc	1585
*E. coli*											
Spring	0 a	0 a	0 a	0 a	23 ab	0 a	8 a	0 a	2 a	0 a	3
Summer	8 a	13 a	0 a	35 ab	27 ab	11 a	15 a	26 ab	12 a	0 a	15
Autumn	10 a	38 ab	0 a	177 c	0 a	32 ab	76 b	48 ab	0 a	0 a	38
Winter	0 a	0 a	0 a	0 a	3 a	0 a	0 a	0 a	0 a	0 a	0

The different letters within a column indicate a significant difference at *p* < 0.05 according to Tukey’s test

*Moderate air contamination described in Table 1

**Heavy air contamination described in Table 1

**Table 4:** Mean number (CFU·m^−3^) of airborne microorganisms—SKH Nielepice

Season	Sampling point				
	1	2	3	4	Mean
Mesophilic bacteria					
Spring	659 a	470 a	297 a	416 a	460
Summer	383 a	374 a	440 a	498 a	424
Autumn	63 a	78 a	65 a	149 a	89
Winter	109 a	45 a	118 a	214 a	122
Mold fungi					
Spring	846 a	1170 a	1056 a	898 a	992
Summer	1241 a	1493 a	1120 a	706 a	1140
Autumn	212 a	280 a	310 a	223 a	256
Winter	104 a	29 a	36 a	36 a	51
Actinomycetes					
Spring	15* a	9 a	1 a	33* a	15
Summer	133** a	143** a	65* a	140** a	120
Autumn	0 a	0 a	0 a	30* a	8
Winter	26* a	7 a	10 a	10 a	13
*Staphylococcus* spp.					
Spring	886 c	347 ab	116 a	198 ab	387
Summer	313 ab	192 ab	256 ab	298 ab	265
Autumn	270 ab	65 a	61 a	55 a	113
Winter	76 a	15 a	34 a	87 a	53

The different letters within a column indicate a significant difference at *p* < 0.05 according to Tukey’s test

*Moderate air contamination described in Table 1

**Heavy air contamination described in Table 1

The limit values for the concentration of bacteria were not exceeded at OJK Pegaz in the control point (5) outside the stables and in points 3 (autumn) and 4 (autumn, winter), which were located in the boxes opening to the outside. In the remaining points, the air was qualified as moderately or heavily polluted with these microorganisms. In OJK Pegaz, bacteria were most abundant in summer (2756 CFU·m^−3^) and their smallest numbers were observed in winter (864 CFU·m^−3^). The concentration of airborne fungi did not exceed the permissible level at any of the sampling points. Their mean concentrations were on the other hand the highest in summer (1202 CFU·m^−3^) and autumn (1000 CFU·m^−3^) but decreased significantly in winter (233 CFU·m^−3^). Large variation in the number of actinomycetes was observed between the sampling points. Therefore, based on their periodic presence in the analyzed sites, the air was classified as clean or moderately or heavily polluted with these microorganisms. Spring was the season of the year with the highest mean number of airborne actinomycetes (271 CFU·m^−3^), whereas winter (13 CFU·m^−3^) and autumn (17 CFU·m^−3^) were the least favorable for their prevalence. During the entire study period, the air collected in the points located within the stables was most heavily polluted with staphylococci. Only in points 3 and 4 (winter, boxes opening to the outside), there was a decrease in the concentration of *Staphylococcus* spp. Moreover, in winter, the average number of staphylococci (370 CFU·m^−3^) was the smallest as compared to the rest of the year. Small numbers of *E. coli* were detected in all sampling points at OJK Pegaz in spring and summer (Table 2).

The limit values for the concentration of mesophilic bacteria were not exceeded at KJK Szary in the control point (10) outside the stables and in the point no. 5, in autumn. In all other cases, the air was classified as moderately or heavily polluted with bacteria. The mean concentration of bacteria at KJK Szary was the largest in summer (2886 CFU·m^−3^) and the smallest in winter (1995 CFU·m^−3^). The concentration of fungi in the air did not exceed the permissible level of contamination in any of the sampling points. Their average concentration was the highest in autumn (2443 CFU·m^−3^) and the smallest in winter (1271 CFU·m^−3^). Large variations in the number of actinomycetes were observed between the sampling points. Therefore, on the basis of their periodic presence in the sampling points, the air was classified as clean or moderately or heavily polluted with these microorganisms. Summer was the season with the highest mean concentration of airborne actinomycetes (1057 CFU·m^−3^), while autumn was the least favorable for these microorganisms (2 CFU·m^−3^) and this was the season in which the presence of actinomycetes was not found in any of the sampling points (except point no. 8). During the entire study period, the samples of air collected from the points situated within the stables were heavily polluted with staphylococci. On the other hand, in the control point (no. 10), outside the stable, their concentration was significantly lower. The highest mean concentration of airborne *Staphylococcus* spp. was observed in summer (2916 CFU·m^−3^) and the lowest in autumn (1680 CFU·m^−3^), and winter (1585 CFU·m^−3^). Small numbers of *E. coli* were detected in most of the sampling points, mostly in summer and autumn (Table 3).

The limit values for the concentrations of airborne bacteria and fungi were not exceeded in SKH Nielepice in any of the sampling points. Therefore, air in SKH Nielepice was classified as uncontaminated with these microorganisms. In contrast, it was found that spring and summer were the seasons in which the mean number of bacteria (460 and 424 CFU·m^−3^, respectively) and fungi (992 and 1140 CFU·m^−3^, respectively) was the highest. Similarly as in other horse riding centers, the numbers of actinomycetes varied largely between the sampling points. Therefore, on the basis of their periodic presence in the sampling points, the air was classified as clean or moderately or heavily polluted with these microorganisms. In summer, the mean concentration of airborne actinomycetes was the highest in all sampling points (120 CFU·m^−3^). During the entire study period, the air samples collected in this horse riding facility was contaminated with staphylococci. However, the numbers of *Staphylococcus* spp. were by the order of magnitude or even two times smaller than in OJK Pegaz and KJK Szary, which was most probably related to the fact that in SKH Nielepice, all sampling points were situated in the open air. Staphylococci in the air of the SKH Nielepice facility were the most abundant in spring (387 CFU·m^−3^). *E. coli* was not detected in any of the sampling points; therefore, these results (0 CFU·m^−3^) were not included (Table 4).

**Fig. 1:**
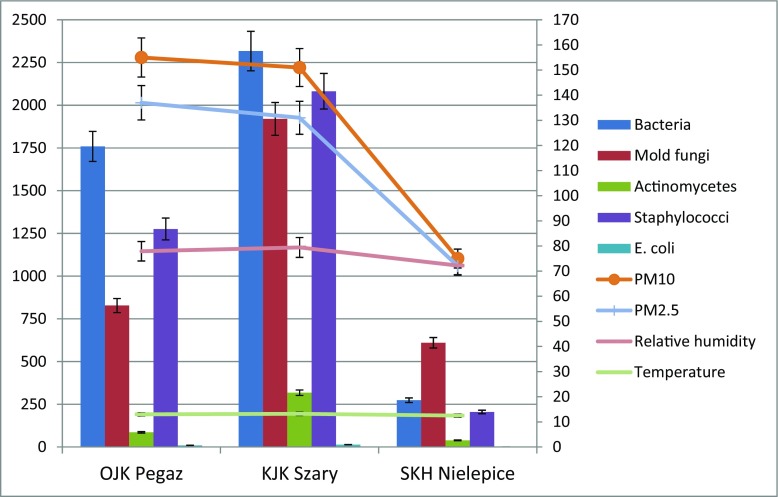
Mean prevalence of the selected microorganisms (CFU·m^−3^), particle pollution (μg · m^−3^), relative humidity (%), and air temperature (°C) in the studied horse riding centers. *The left axis is assigned to bioaerosol; the right axis is assigned to microclimatic parameters

Figure 1 shows mean numbers of the examined microbial groups in the studied horse riding centers over 3 years of the study. Based on the obtained results, it can be concluded that the total number of all examined microorganisms was the highest in KJK Szary, followed by OJK Pegaz and SKH Nielepice.

Regarding the seasonal variation, Budzińska et al. (2016) observed varying concentrations of airborne bacteria in stables—from 1.04·10^5^ CFU·m^−3^ in autumn to 5.90·10^5^ CFU·m^−3^ in summer. On the other hand, the smallest number of bacteria was reported in winter, i.e., 3.74·10^4^ CFU·m^−3^. In the study by Samadi et al. (2009), the numbers of bacteria in the air of stables ranged from 1.22·10^3^ to 7.82·10^3^ CFU·m^−3^. Dutkiewicz et al. (1994) observed the number of bacteria in the air of stables at the levels varying from 2.6·10^4^ to 1.5·10^5^ CFU·m^−3^. The concentration of mold fungi in this study did not exceed the limit values included in the Polish Standards (PN-89/Z-04111/03). Budzińska et al. (2016) reported that the concentration of fungi in stables ranged from 1.27·10^3^ CFU·m^−3^ in winter to 4.78·10^4^ CFU·m^−3^ in autumn. On the other hand, the range of concentrations of airborne fungi observed by Witkowska et al. (2012) was 10^3−^10^4^ CFU·m^−3^ and Dutkiewicz et al. (1994) observed the concentrations within the values of 1.7·10^3^–2.8·10^4^ CFU·m^−3^. Prevalence of these microorganisms is strongly associated with the season of the year, air temperature, stocking density, ventilation system, and the hygiene in stables (Dutkiewicz et al. 1994; Budzińska et al. 2016). High concentrations of airborne molds in the stables were also demonstrated by Nardoni et al. (2005), who reported their values reaching from 1750 to 3000 CFU·m^−3^. Actinomycetes were very abundant in the study by Budzińska et al. (2016), from 3.52·10^3^ CFU·m^−3^ in summer to even 9.03·10^3^ CFU·m^−3^ in spring. Elfman et al. (2009) reported that actinomycetes were very abundant in the air of stables, which was related to the high concentrations of organic dust suspended in the air, affecting their concentrations. At the same time, Elfman et al. (2009) indicated that the highest number of actinomycetes is typical of February and March. In the study by Budzińska et al. (2016), mannitol-positive staphylococci were isolated from the air of stables in the numbers ranging from 0.52·10^1^ CFU·m^−3^ in winter to 3.94·10^1^ CFU·m^−3^ in summer, whereas the numbers of hemolytic staphylococci ranged from 0.43·10^1^ CFU·m^−3^ in spring to 8.62·10^2^ CFU·m^−3^ in summer. Korzekwa et al. (2015) found the lowest mean prevalence of staphylococci in winter—at the level of 157 CFU·m^−3^—and the highest in summer—16,008 CFU·m^−3^. In summer and autumn, Budzińska et al. (2016) observed the highest numbers of airborne staphylococci, which are the most frequently detected bacteria in livestock premises (Dutkiewicz et al. 1994). Budzińska et al. (2016) detected bacteria from the family of *Enterobacteriaceae* in the air of stables at the levels from 1.38·10^2^ CFU·m^−3^ in winter to 1.02·10^3^ CFU·m^−3^ in summer. The presence of airborne *Enterobacteriaceae* and particularly *E. coli* is a cause for concern because it is an epidemiological threat to both humans and animals (Budzińska et al. 2016). According to Zucker et al. (2000), *E. coli* is an often isolated bacterium from the air of livestock premises. Also, according to Zucker et al. (2000) and Lloyd et al. (2003), airborne *E. coli* in livestock premises originates from manure.

As shown by other authors (Nardoni et al. 2005; Elfman et al. 2009; Samadi et al. 2009; Witkowska et al. 2012; Korzekwa et al. 2015; Budzińska et al. 2016), the concentration of microorganisms in the stables is subject to changes and is dependent on a number of factors, i.e., microclimatic conditions, temperature, relative humidity, particulate matter concentration, ventilation system, and stocking density. In addition, Witkowska et al. (2012) and Elfman et al. (2009) reported that the number of microorganisms in the air of stables was lower and similar to one another in spring and autumn, while it increased in summer and winter. Witkowska et al. (2012) explained this situation by the fact that in winter, the ventilation in the stables is limited to the minimum, due to the loss of heat; thus, the concentration and survival of microorganisms in the air are higher. On the other hand, in summer, the increase in the number of microorganisms is affected by the higher air temperature.

Tables 5, 6, and 7 show mean values of the following parameters: air temperature, particulate matter concentration (PM_10_ and PM_2.5_), and relative humidity taking into consideration the seasons of the year and sampling points. The analysis of temperature changes in OJK Pegaz in the analyzed period allowed concluding that there are only small differences in the temperature between the points situated within the stable (no. 1–4) and the control point (no. 5), located outside. Only in the winter, at the control point, the air temperature was below zero and therefore differed significantly from the one observed in the remaining points within the stable. The highest temperature amplitude between the analyzed sampling points, i.e., of 2.4 °C, was found in the winter. Relative humidity was the highest in autumn and winter, and it was observed that in the control point (5), it reached the lowest values in all seasons of the year. The mean values of PM_10_ concentration during all measurements fall within the range from 49 μg·m^−3^ (summer, control point (5)) to 748 μg·m^−3^ (winter, closed box stall (1)), which corresponds to 98–1496% of the permissible concentration, i.e., 50 μg·m^−3^ (Regulation 2012). The mean concentrations of PM_2.5_ during all measurements ranged from 48 μg·m^−3^ (summer, box opening to the outside (3), control point (5), and autumn, box opening to the outside (4)) to 654 μg·m^−3^ (winter, closed box stall (1)), which corresponds to 192–2616% of the permissible concentration, i.e., 25 μg·m^−3^ (Regulation 2012). The PM_2.5_ concentration exceeded the permissible values in all sampling points during the entire study period. Analysis of the particulate matter concentration in terms of both PM_10_ and PM_2.5_ showed that winter was the season of the year when the transgressions of limits specified in the Regulation (Regulation 2012) were the highest.

**Table 5:** Mean temperature, relative humidity, and particulate matter concentrations—OJK Pegaz

Sampling point	Temperature (°C)				Relative humidity (%)			
	Spring	Summer	Autumn	Winter	Spring	Summer	Autumn	Winter
1	15.3	23.9	12.4	2.8	81.2	63.9	86.1	85.4
2	15.4	23.6	11.6	3	83.9	66	91	86.8
3	14.4	24.3	10.1	3	75.2	70.1	81.1	83.5
4	14.9	23.4	10.5	2.9	80.4	65.4	84.4	85.7
5	14.5	24.8	10.3	− 0.6	70.9	59.4	82.1	75.3
Mean	14.9	24	11	2.2	78.3	65	84.9	83.3
Sampling point	PM_10_ (μg m^−3^)				PM_2.5_ (μg m^−3^)			
	Spring	Summer	Autumn	Winter	Spring	Summer	Autumn	Winter
1	98*	97*	104*	748*	75*	69*	83*	654*
2	105*	97*	73*	714*	92*	78*	64*	647*
3	55*	50	55*	185*	53*	48*	50*	166*
4	64*	61*	56*	192*	56*	50*	48*	184*
5	72*	49	51*	172*	61*	48*	49*	157*
Mean	79	71	68	402	67	59	59	362

*Limit values provided in the Regulation of the Minister of the Environment of 24 August 2012 on the levels of certain substances in the air (PM_10_ > 50 μg m^−3^ and PM_2.5_ > 25 μg m^−3^)

**Table 6:** Mean temperature, relative humidity, and particulate matter concentrations—KJK Szary

Sampling point	Temperature (°C)				Relative humidity (%)			
	Spring	Summer	Autumn	Winter	Spring	Summer	Autumn	Winter
1	12.5	23.7	13	2.4	78.9	67.3	85.4	91.6
2	13.1	22.4	13.4	3.1	85.9	68.1	87.1	90.2
3	13.3	22.3	13.7	3.7	76.6	71.2	85.3	87.7
4	13.5	22.6	13.8	3.2	82.5	66.4	83.7	87.7
5	14.4	22.2	13.6	4.1	82.6	67.6	88.6	87.4
6	14.3	22.6	13.9	3.7	84.8	67	87	87.7
7	12.8	22.3	13.4	3.2	74.6	74.1	82	87
8	13.1	21.7	13.5	3.6	75.9	62.4	79.8	90.9
9	13.5	22.3	13.8	3.7	80.7	67.1	85.5	85.9
10	13.2	23.5	14.5	1	72.4	63.5	74.9	72
Mean	13.4	22.6	13.7	3.2	79.5	67.5	83.9	86.8
Sampling point	PM_10_ (μg m^−3^)				PM_2.5_ (μg m^−3^)			
	Spring	Summer	Autumn	Winter	Spring	Summer	Autumn	Winter
1	88*	135*	82*	372*	68*	84*	75*	364*
2	93*	78*	93*	391*	77*	69*	78*	382*
3	97*	100*	110*	406*	82*	80*	92*	395*
4	91*	100*	74*	336*	63*	76*	69*	305*
5	76*	90*	76*	506*	63*	68*	68*	408*
6	119*	98*	97*	296*	78*	77*	90*	261*
7	101*	69*	104*	299*	80*	62*	95*	272*
8	148*	94*	102*	282*	88*	75*	88*	245*
9	87*	73*	91*	225*	73*	66*	87*	203*
10	72*	71*	74*	136*	63*	64*	68*	118*
Mean	97	91	90	325	74	72	81	295

*Limit values provided in the Regulation of the Minister of the Environment of 24 August 2012 on the levels of certain substances in the air (PM_10_ > 50 μg m^−3^ and PM_2.5_ > 25 μg m^−3^)

**Table 7:** Mean temperature, relative humidity, and particulate matter concentrations—SKH Nielepice

Sampling point	Temperature (°C)				Relative humidity (%)			
	Spring	Summer	Autumn	Winter	Spring	Summer	Autumn	Winter
1	13	22.8	13.6	− 0.1	71.1	58.8	80.3	74.4
2	12.9	23.9	13.4	− 0.1	73.5	65.2	78.8	69.4
3	12.9	23.9	13.5	− 0.3	73.4	65.9	77	76.7
4	13	23.5	13.3	− 0.3	72	68.3	80.2	69.4
Mean	13	23.5	13.5	− 0.2	72.5	64.6	79.1	72.5
Sampling point	PM_10_ (μg m^−3^)				PM_2.5_ (μg m^−3^)			
	Spring	Summer	Autumn	Winter	Spring	Summer	Autumn	Winter
1	87*	53*	103*	78*	82*	52*	106*	73*
2	76*	50	87*	84*	72*	48*	83*	79*
3	73*	48	74*	89*	70*	47*	72*	83*
4	77*	50	86*	90*	74*	48*	82*	76*
Mean	78	50	88	85	75	49	86	78

*Limit values provided in the Regulation of the Minister of the Environment of 24 August 2012 on the levels of certain substances in the air (PM_10_ > 50 μg m^−3^ and PM_2.5_ > 25 μg m^−3^)

Analysis of temperature changes in KJK Szary during the analyzed period allowed concluding that there are also only small differences in the temperature between the points situated within the stable (no. 1–9) and the control point (10), situated outside. The highest temperature amplitude between the analyzed sampling points, i.e., of 3.1 °C, was found between the control point (10), in the open air, and the point no. 5 located within the stable. The temperature in all sampling points was above zero. Relative humidity was the highest in the autumn and winter, and it was observed that in the control point (no. 10), it was the lowest in all seasons of the year. The mean values of PM_10_ concentration during all measurements ranged from 71 μg·m^−3^ (summer, control point (10)) to 506 μg·m^−3^ (winter, box (5)), which corresponds to 142–1012% of the permissible concentration of 50 μg·m^−3^ (Regulation 2012). The mean concentrations of PM_2.5_ during all measurements ranged from 62 μg·m^−3^ (summer, box (7)) to 408 μg·m^−3^ (winter, box (5)), which corresponds to 248–1632% of the permissible concentration of 25 μg·m^−3^ (Regulation 2012). The concentrations of PM_10_ and PM_2.5_ exceeded the permissible levels in all sampling points during the entire study period. Analysis of the particulate matter concentration in terms of both PM_10_ and PM_2.5_ showed that winter was the season of the year when the transgressions of limits specified in the Regulation (Regulation 2012) were the highest.

Analysis of temperature changes in SKH Nielepice during the analyzed period allowed concluding again that there are small differences between sampling points situated within the stable. The highest temperature amplitude between the analyzed sampling points—of 1.1 °C—was found in summer between the points no. 1 (roof for riders) and 2 (saddle room), and 3 (roof for horses). In the winter, the temperature in all sampling points was below zero. Relative humidity was the highest in autumn, with no significant variation in its values between the sampling points in different seasons of the year. The mean values of PM_10_ concentration during all measurements ranged from 48 μg·m^−3^ (summer, roof for horses (3)) to 103 μg·m^−3^ (autumn, roof for riders (1)), corresponding to 96–206% of the permissible concentration of 50 μg·m^−3^ (Regulation 2012). The mean concentrations of PM_2.5_ during all measurements ranged from 47 μg·m^−3^ (summer, roof for horses (3)) to 106 μg·m^−3^ (autumn, roof for riders (1)), corresponding to 188–424% of the permissible concentration, i.e., 25 μg·m^−3^ (Regulation 2012). The concentration of PM_2.5_ exceeded the permissible limits in all sampling points, during the entire study period. Analysis of the particulate matter concentration in terms of both PM_10_ and PM_2.5_ showed that autumn and winter were the seasons of the year when the transgressions of limits specified in the Regulation (Regulation 2012) were the highest.

The air temperature recorded during the study period did not differ from the values typical for particular seasons of the year in this region of Poland. The temperature measured inside the stables and outside the buildings due to good ventilation of the facilities was often similar. The temperature values in stables measured by Bombik et al. (2011) fell within the range of 8.5–14.4 °C and outside the buildings 7.4–14.8 °C. On the other hand, Kwiatkowska-Stenzel et al. (2011), while comparing microclimatic conditions in the box-stall stable and in the run pen, found the temperatures within the ranges − 0.9–15.5 °C and − 7.1–13.4 °C, respectively. Budzińska et al. (2016), depending on the season of the year, recorded the following temperatures in the stables: spring 12.4 °C, summer 21.2 °C, autumn 12.6 °C, and winter 8.2 °C. In this study, temperature was the factor that most significantly affected the prevalence of microorganisms in the air of the tested stables. Higher numbers of microorganisms were recorded in the seasons of the year, when higher temperatures are typical.

In the study by Bombik et al. (2011), relative humidity in the stables was 50.7–83% and outside the buildings—51–89.1%. Budzińska et al. (2016), depending on the season of the year, recorded the following values of relative humidity in the stables: spring 65.4%, summer 60.2%, autumn 74.6%, and winter 76.2%. On the other hand, Kwiatkowska-Stenzel et al. (2011), while comparing microclimatic conditions in the box-stall stable and in the run pen, found the relative humidity of 91.8 and 94%, respectively.

According to the aerodynamic diameter of the particles, particulate matter is classified into two main fractions: PM_10_ and PM_2.5_, which air quality monitoring in Poland and Europe refers to (Regulation 2012; Marcazzan et al. 2001). The transgressions of PM_10_ and PM_2.5_ limit values, recorded in this study, are due to both smog occurring in Kraków and the specific character of the examined livestock premises (Pawul and Śliwka 2016).

Based on the statistical analysis of the obtained results, it was found that in all three horse riding centers, the correlation between the abundance of selected groups of microorganisms and microclimate conditions (temperature and humidity of air, PM_10_, and PM_2.5_) was positive in the case of temperature and negative for the other analyzed factors. This is probably due to the fact that in the winter months due to low temperatures, the number of microorganisms was lower, even though the general particulate matter concentration was greater. This is most probably caused by the occurrence of smog phenomenon in this region of Poland, which becomes particularly troublesome during the heating season. However, the analysis showed a significant correlation between the concentration of bioaerosol and temperature (for bacteria: *r* = 0.99; *p* < 0.05), of bioaerosol and particulate matter—both fractions PM_10_ and PM_2.5_ (for mold fungi: *r* = − 0.95, *Staphylococcus* spp.: *r* = − 0.96; *p* < 0.05) in OJK Pegaz, a significant correlation between the concentration of bioaerosol and air humidity (both for bacteria and *Staphylococcus* spp.: *r* = − 0.99; *p* < 0.05) in KJK Szary, a significant correlation between the concentration of bioaerosol and particulate matter—fractions PM_10_ and PM_2.5_ (for actinomycetes accordingly: *r* = − 0.98, *r* = − 0.97; *p* < 0.05) in SKH Nielepice.

As shown in Fig. 1, air pollution with both fractions of particles was clearly higher in OJK Pegaz and KJK Szary than in SKH Nielepice. In the case of temperature and relative humidity values, differences between horse riding centers are not so evident. It is suggested that this situation results from the differences in the horse keeping systems. In the centers that run the box-stall system of horse keeping, the animals spend a lot of time in their boxes. Many care and cleaning operations are carried out on a limited, closed space. In addition, there is manure in the boxes and there are feed, straw, and hay for horses in the farmhouses, constituting the source of particulate pollution of air (Fleming et al. 2008). Although the KJK Szary is a modern and well-ventilated facility, the PM_10_ and PM_2.5_ concentration was comparably high to the one recorded in smaller and older facility, which is OJK Pegaz. Nevertheless, it should be added that the high concentrations of particulate matter, also in SKH Nielepice, were also the result of generally poor air quality in Lesser Poland, where the problem of air pollution has been observed for many years (Pawul and Śliwka 2016). This is particularly evident during the heating season, which is winter in the Polish climate. Unfortunately, many fireplaces burn low-quality fuel, and sometimes even garbage and waste. Therefore, the problem of particle pollution in the horse riding centers should be perceived in two ways, i.e., as the effect of generally bad air quality but also as the specific characteristic of the livestock premises. It is justified in the stables to undertake activities aimed at reducing the PM_2.5_ and PM_10_ dust content and the bioaerosol concentration. It is necessary to remove manure every day and replace bedding with a new one. In addition, straw, hay, and fodder should be stored in separate, specially designed premises. Daily airing of the stable in the absence of mechanical ventilation is also a good concept. All care treatments, i.e., horse haircut and brushing, should be performed in specially designated area or outside the stables. This approach can contribute to improving the air quality in the stable and have a positive impact on the health of horses and people working with them.

## Conclusions

On the basis of the results obtained, it can be concluded that the air pollution is higher in the horse riding centers with the box-stall horse keeping system. Particularly high transgressions of the limit values were observed in the largest analyzed facility—KJK Szary. In addition, the concentration of bioaerosol also depended on the temperature and humidity of air and was subject to seasonal fluctuations. High concentration of particulate matter PM_10_ and PM_2.5_ occurring mostly in the facilities with the box-stall horse keeping system—KJK Szary and OJK Pegaz—is also the reason for the increase of microbial air pollution. The presence of airborne *E. coli*, *Staphylococcus* spp., actinomycetes, and mold fungi, observed in this study, and therefore also their metabolites and toxins, can cause illness in both horses and people staying around them. Therefore, regardless of the horse keeping system, the conditions in which the animals stay must be optimal and tailored to their needs in order to maintain them in good health and condition and to prevent the exposure of people who stay around them to the harmful effects of bioaerosol.
